# Socioeconomic inequalities in distance to and participation in a community-based running and walking activity: A longitudinal ecological study of parkrun 2010 to 2019^☆^^☆☆^

**DOI:** 10.1016/j.healthplace.2021.102626

**Published:** 2021-09

**Authors:** Robert A. Smith, Paul P. Schneider, Rami Cosulich, Helen Quirk, Alice M. Bullas, Steve J. Haake, Elizabeth Goyder

**Affiliations:** a School of Health and Related Research, 30 Regents Court, Sheffield, S1 4DA, UK; b Advanced Wellbeing Research Centre, Sheffield Hallam University, Olympic Legacy Park, Sheffield, S9 3TU, UK; c Sports Engineering, Sport and Physical Activity Research Centre, Sheffield Hallam University, S10 2LW, UK

**Keywords:** Parkrun, Physical activity, Socioeconomic deprivation, Ecological study, Relative index of inequality

## Abstract

**Objectives:**

To conduct a longitudinal ecological analysis of the distance to and participation in free weekly outdoor physical activity events (*parkrun*) in England from 2010 to 2019, and related socioeconomic and ethnic inequalities, to inform policies to support participation in physically active community events.

**Methods:**

We calculate distance to the nearest *parkrun* event for each English Lower Layer Super Output Area (LSOA) each month from January 2010 to December 2019. We then report the trends in distance to and participation in *parkrun* by Index of Multiple Deprivation quintile. We also report trends in the Relative Index of Inequality (RII) by deprivation for participation and distance to nearest event. We go on to investigate trends in LSOA level determinants (e.g. deprivation and ethnic density) of *parkrun* participation between 2010 and 2019, using multivariable Poisson regression models.

**Results:**

Mean distance to the nearest *parkrun* event decreased from 34.1 km in 2010, to 4.6 km in 2019. Throughout the period, *parkrun* events tended to be situated closer to deprived areas compared to less deprived areas. Participation rates increased superlinearly (greater than linear increase) from 2010 to 2013 before slowing to linear growth. Participation over the period exhibits a clear socioeconomic gradient, with people from deprived areas having consistently lower participation rates over the period. *parkrun* participation rates became more equal between 2010 and 2013 (RII improved from 189 to 39), before stabilising at an RII between 32.9 and 39.6 from 2014 to 2019. The results of the Poisson regression model validate this finding; the coefficients on IMD score initially increased from −0.050 in 2010 to −0.038 in 2013, and then remained relatively stable to 2019 (−0.035).

**Conclusions:**

Over the past 10 years, geodesic distance to the nearest *parkrun* decreased from a mean of 34 km to 5 km. In 2010, there was equality between the least and most deprived areas but by 2017 the distance of the most deprived areas was 29% that of the least deprived. Participation was shown to have increased over the past 10 years which can be split into two distinct phases: from 2010 to 2013 participation increased super-linearly and inequality in participation fell dramatically; from 2013 to 2019 participation increased linearly, and inequality in participation remained stable. Despite *parkrun*'s ambitions of creating inclusive events and engaging with deprived communities, the socioeconomic gradient in participation rates remained high and stable since 2013. Gaining a better understanding of the reasons why *parkrun* grew so quickly may be useful for other physical activity movements, while further analysis of the relatively lower participation rates in areas with higher socioeconomic deprivation is important for developing initiatives to encourage physical activity in these communities.

## Highlights

• Geographical access to & participation in parkrun in England improved every year from 2010 to 2019.
• However, the rate of improvement slowed down, especially after 2013.
• New events reduced distance to parkrun in high deprivation areas more than low deprivation areas.
• Socioeconomic inequalities in participation fell from 2010 to 2013 and stabilised after.

## 1 Introduction

Physical activity follows a socioeconomic gradient (Stalsberg and Pedersen, 2018). People from lower socioeconomic status groups participate in less leisure time physical activity and organised physical activity compared to higher socioeconomic status groups (Stalsberg and Pedersen, 2018; Farrell et al., 2014). This has been attributed to various barriers including, but not limited to: time available to participate, work patterns, costs of participation, low awareness, higher stress, crime rates in the local area, perceived safety, lower self-efficacy and lower social support (Pampel et al., 2010; Cerin and Leslie, 2008; Withall et al., 2011; Jones et al., 2009). Thus, in the promotion of active lifestyles, consideration of individual characteristics and attributes of the physical environment are important (Stalsberg and Pedersen, 2018; Farrell et al., 2014; Jones et al., 2009). However, provision of a supportive environment for being active (facilities, spaces and ways to be active) might not always be equal (Panter et al., 2008).

In England, a negative association has been found between neighbourhood deprivation and density of physical activity facilities, with more deprived areas having poorer access (Hillsdon et al., 2007). Whilst observations from the cities of Glasgow and Bristol (UK) have found that the proportion of the population living nearer greenspace in which to be physically active is greater among the more deprived (Jones et al., 2009; Fairburn et al., 2005), we also know that more economically deprived areas have less available good quality greenspace (Ridgley, 2020).

Physical activity policies and activity organisers must commit to addressing equitable and inclusive access to physical activity resources (Hämäläinen et al., 2016; Ridgley, 2020). Robust evaluation of participation and access to organised activities would ensure that health inequalities are not inadvertently exacerbated. One example of a physical activity initiative taking place in public spaces across the UK and worldwide is *parkrun*. *parkrun* is a charity that organises free, weekly, timed 5-km outdoor events in the community for people aged 4 and above to participate as runners, walkers or volunteers. It has over 2000 events in 22 countries worldwide. At the time of writing 6.3 million people have taken part, many of whom were not previously engaged in walking or running, or even physically active, prior to *parkrun* (Reece et al., 2019; Stevinson et al., 2015; Haake et al., 2018). Early research showed that regular participants in *parkrun* experienced increases in weekly physical activity levels, improved fitness, and reported health benefits such as better weight control and mental wellbeing (Stevinson et al., 2015). This has led to *parkrun* being identified as an exemplar intervention in the WHO Global Action Plan on Physical Activity 2018–2030 (World Health Organization, 2019), and by the Royal College of General Practitioners (RCGP) as a form of social prescribing aimed at increasing patient physical activity (Fleming et al., 2020).

As a grass-roots, citizen led community organisation, *parkrun*s are established by enthusiastic volunteers in their local community (Wiltshire and Stevinson, 2018). As a result, there is a risk that, as with other public health interventions, *parkrun* events may not be as available, or as well attended by people living in deprived areas as in less deprived areas (Bull et al., 2014). In 2018, Sport England announced funding to help create 200 new *parkrun* events in England, with a core aim to improve participation among women and girls and those from socioeconomically deprived areas. Previous studies used determinants of access to and participation in *parkrun* (Smith et al., 2020; Schneider et al., 2020) to determine the optimal location of these new events. This body of work showed that, despite similar geographical access to *parkrun* events, people from more deprived areas and areas with higher ethnic density had lower participation rates than less deprived areas with lower ethnic density. However, as a cross-sectional study with data from a single year, it was not possible to understand how access to and participation in *parkrun* had changed over time (Schneider et al., 2020). Therefore, the UK organisers of *parkrun*, *parkrunUK*, made additional data available with the specific objective of improving understanding of the trends in geographic access and participation. Our research question for this paper is therefore: how have the determinants of geographical distance to, and participation in, *parkrun* changed over the period from 2010 to 2019?

This paper aims to investigate the trends in distance to, and participation in, *parkrun* events between 2010 and 2019 in relation to socioeconomic deprivation and ethnic density. We utilise rich datasets from *parkrunUK* and the Office for National Statistics (ONS), including the weekly number of *parkrun* finishers from each of the 32,844 Lower layer Super Output Areas (LSOAs) in England over the ten-year period from 2010 to 2019.

## 2 Methods

### Ethical statement

Ethical approval was obtained from the Sheffield Hallam University Ethics Committee (ER10776545). We did not collect any personal information, but only used aggregate secondary data at the Lower layer Super Output Area level from *parkrun* and publicly accessible data from the Office for National Statistics. It is therefore not possible to identify individuals. The *parkrun* Research Board approved this research project.

### 2.1 Data sources and variables

Data on the number of finishers from each of the 32,844 LSOAs on the 522 Saturdays in the years from 2010 to 2019 inclusive was obtained from *parkrunUK*. The geographical location and start date for each *parkrun* event was obtained from the *parkrunUK* website. The rest of the data was obtained from the ONS. Descriptions of variables and sources are provided in Table 1 below. All underlying data is provided open source (https://github.com/RobertASmith/parkrun_temporal). In the open source data, the number of finishers is provided aggregated by month, as used in the remainder of the analysis.

**Table 1:** Variables & sources of data in analysis.

Variable	Description	Source
run_count	number of finishers per month/year	*parkrunUK*
run_rate	derived from run_count and LSOA populations	derived
imd	Index of Multiple Deprivation scores for each LSOA	ONS, 2015
total_pop	total number of individuals in each LSOA	ONS, 2017
pop_density	population density for each LSOA	ONS, 2017
ethnic_density	Ethnic Density: percentage of population non-White-British	ONS, 2011
mn_dstn	distance (in km) from LSOA centroid to nearest *parkrun* (derived)	derived from ONS. 2011

For each of the 32,844 LSOAs, we computed the geodesic (i.e. direct linear) distances between its population-weighted centroid and all *parkrun* events that were in operation on the 15th of the respective month, and then selected the shortest distance. We took this as a proxy for access to *parkrun* events, whereby a shorter distance to the nearest event was assumed to reflect ‘better access’. The limitations of this are outlined in the discussion.

Participation for a given LSOA was defined as the number of times anyone living in the LSOA finished a *parkrun* event in England in the respective time-period (month or year depending on the analysis). Four finishers could therefore be the result of one individual finishing four events, or four individuals finishing one event each.

The socioeconomic deprivation of LSOAs was measured using the 2015 Index of Multiple Deprivation (IMD), a measure of relative deprivation. The IMD combines into a single score 37 indicators from seven domains (income, employment, education and skills, health and disability, crime, housing and services, and living environment). The score ranges from 0 (least deprived) to 100 (most deprived). We only used IMD data from 2015, as IMD data is not comparable across different years and the ONS does not recommend constructing time-series by interpolating IMD estimates.

Ethnic Density, the percentage of the population reporting as non-White-British, was estimated as 100 minus the percentage reporting as White British and was obtained from the ONS dataset (Table 1).

### 2.2 Data analysis

The open source dataset contains data for all 32,844 English LSOAs for each month from January 2010 to December 2019, including only events which took place on Saturdays. As an ecological study, all analyses are conducted at the level of the LSOA, and results are not weighted by population size.

#### 2.2.1 Descriptive statistics

We investigate longitudinal trends in geodesic distance to nearest event location and participation by IMD quintile using descriptive statistics and charts. We report both the number of finishers per 1000 persons and the mean distance to nearest event for each of the IMD quintiles by month and year.

#### 2.2.2 Relative index of inequality

The relative index of inequality (RII) is a strictly non-negative regression-based index which is commonly used to describe the size of the effect of socioeconomic status on an outcome (Mondor et al., 2018). It is the ratio of the predicted outcome in the least deprived area compared to the predicted outcome in the most deprived area. Since it is a regression-based index it takes account of all the data-points and is less sensitive to outliers at the extremes. A RII of 1 indicates no socioeconomic gradient in the outcome of interest; a value higher than 1 indicates a higher predicted value for less deprived groups, while a RII lower than 1 indicates a higher predicted value for more deprived groups.

We calculate the RII, for both geodesic distance to nearest event and participation. The RII for geodesic distance was computed as the ratio of the predicted distance to the nearest *parkrun* event for the least compared to the most deprived LSOA, using a linear regression model with IMD as the only predictor. The RII for participation was calculated as the ratio of the predicted number of finishers from the least compared to the most deprived LSOA, using a univariable Poisson regression model with a log link with total population as the offset variable. For geodesic distance, an RII >1 indicates that less deprived areas are further from their nearest *parkrun*, while for participation an RII >1 indicates that less deprived areas have higher *parkrun* participation rates.

#### 2.2.3 Determinants of participation over time

We replicate our previous analysis of the determinants of community level *parkrun* participation, using a log-link Poisson regression model for aggregate data for each year from 2010 to 2019. As independent variables we use IMD score, population density, ethnic density, and distance to nearest event (in km). Total population was used as an offset. We report mean coefficient estimates and standard errors for each year. We also conducted a stratified analysis, investigating the effects of the covariates in rural and urban areas separately.

All analyses were undertaken in R version 4.0.2 (2020-06-22). All code is available open source online here: (Anonymous).

## 3 Results

### 3.1 Descriptive statistics

Table 2 shows a summary of the monthly dataset which contains 3,547,152 rows, one row for each unique LSOA each month with a mean IMD score of 21.7 (IQR = 9.6–30.0), mean total population 1666 (IQR = 1437–1750), mean ethnic density of 13.8% (IQR = 2.3%–16.7%), mean population density of 4423 persons per square kilometre (IQR 1288–5924) and mean distance to nearest event of 12.2 km (IQR 2.9 km–13.4 km).

**Table 2:** Descriptive Statistics for time invariant LSOA characteristics (N = 32,844).

Variable	Mean	Min	Pctl(25)	Median	Pctl(75)	Max
IMD score	21.7	0.5	9.6	17.4	30.0	92.6
Ethnic density (%)	13.8	0	2.3	5.2	16.7	99.3
Population	1666	934	1437	1572	1750	7976
Pop density (pop/km^2^)	4423	2.5	1288	3551	5924	99,024

**Fig. 1:**
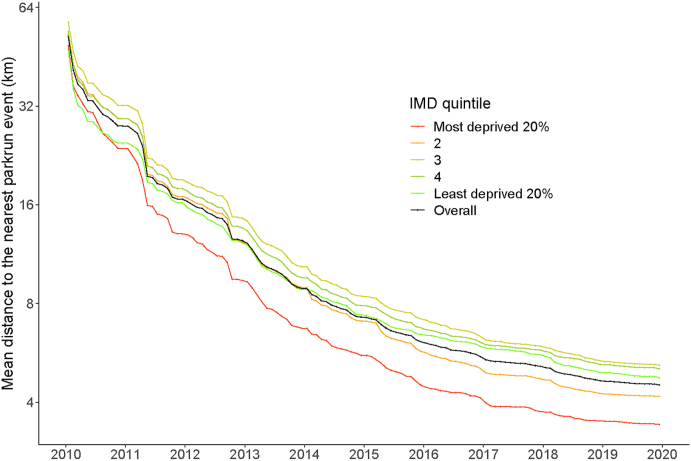
Mean geodesic distance (in km) to nearest event from Jan2010 to Dec 2019, by IMD quintile. See also Table A1.

Fig. 1 shows the mean geodesic distance to the nearest *parkrun* event for each of the IMD quintiles (and overall in black) over time. A table containing the numeric values can be found in Table A1 in the appendix. The mean distance decreased super-linearly in the first four years (from 34 km in 2010 to 10 km in 2013), and took another six years to reduce to less than 5 km. This effect is notable in all IMD quintiles. Geodesic distance to the nearest event had no clear socioeconomic gradient from 2010 to 2013, but from 2013 to 2019 was smaller in the most deprived quintiles (Fig. 1).

**Fig. 2:**
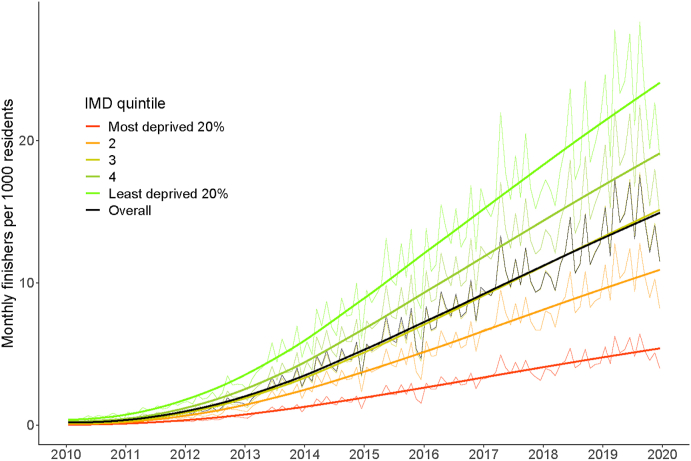
Mean monthly *parkrun* finishers per 1000 persons from Jan2010 to Dec 2019, by IMD quintile. See also Table A2.

Fig. 2 shows the number of finishers per 1000 persons for each IMD quintile, and overall, for each month in the study period. A table of the numeric values can be found in Table A2 in the appendix. The participation rate showed a general positive trend (ignoring seasonal fluctuations) in all deprivation quintiles. In all cases participation can be seen to increase exponentially from 2010 to 2013, before exhibiting linear growth from 2014 to 2019.

There is a clear difference between the participation rates for different IMD deprivation quintiles, with the least deprived quintile having between 4.5 and 7 times the *parkrun* participation rates of the most deprived quintile. By 2019 the most deprived quintile of the population had similar participation rates as the least deprived did in 2013, six years earlier (approximately 5 finishers per 1000).

### 3.2 Trends in relative index of inequality in distance to nearest event

**Fig. 3:**
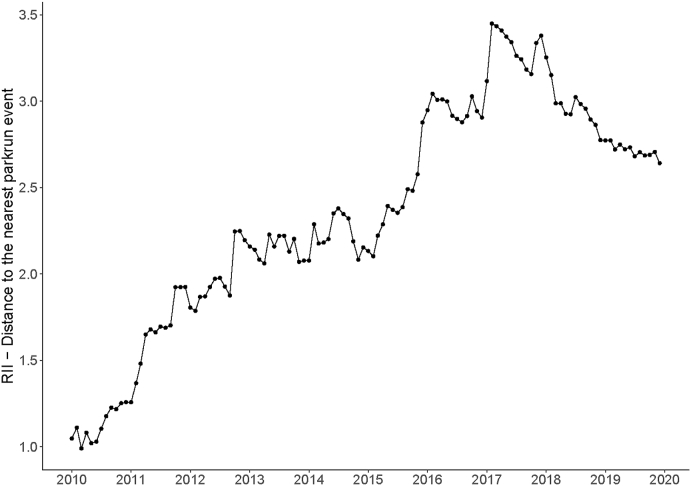
RII in geodesic distance to nearest *parkrun* event by month from Jan10 to Dec19.

Fig. 3 shows the RII for distance, measured as the geodesic distance (in km) to the nearest *parkrun* event, over the period. An RII >1 means that less deprived areas had greater geodesic distances to their nearest *parkrun* event than more deprived areas. We can see that the distance to nearest event was equal in 2010 (i.e. RII 1) when the overall mean distance was 34.1 km (Table A1) but became increasingly unequal such that shorter distances were found for more deprived areas until 2017 where distances for the least deprived areas were almost 3.5 times those of the most deprived LSOA. Put another way, the distance of the most deprived areas from their nearest *parkrun* was 29% of that for the least deprived. By this time, the mean distance was 5.3 km. RII dropped to approximately 2.71 by the end of 2019 when mean distance was 4.6 km (Table A1).

**Fig. 4:**
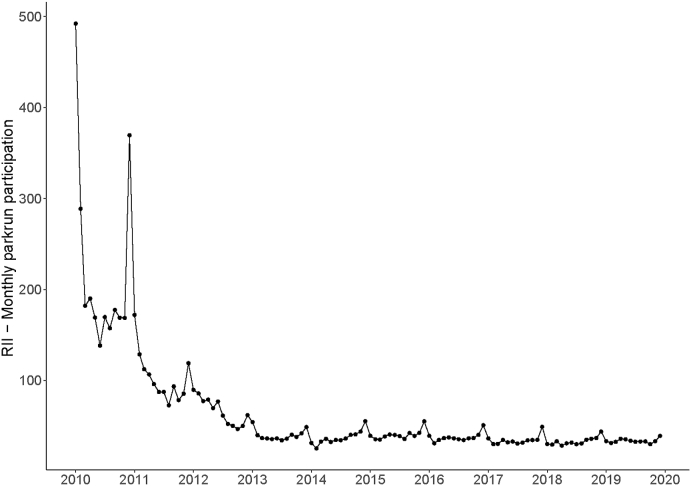
RII in *parkrun* participation by month from Jan10 to Dec19.

Fig. 4 shows the RII for monthly *parkrun* participation. An RII >1 means that less deprived areas had higher participation rates than more deprived areas. Initially in 2010, the socioeconomic gradient of *parkrun* participation was extremely steep, regression-based predictions of participation rates (RII) were 189 times higher in the least deprived LSOA compared to the most deprived LSOA. Subsequently, the RII fell from 2010 to 2013, at which point the measure stabilised such that the least deprived area had around 35 times the predicted number of finishers as the most deprived area. We also found that the relationship exhibited yearly seasonality from the year 2013 onwards, with December having the highest RII value and January the lowest.

### 3.3 Poisson regression model of the determinants of participation from 2010 to 2019

The results of the Poisson regression models, one for each year from 2010 to 2019, are displayed in Table 3. The dependent variable is number of finishers per year, and the independent variables include the LSOA IMD score, ethnic-density (%), population density^1^ and distance to nearest event (in km).

**Table 3:** Results of the Poisson log-link generalised linear model for each year from 2010 to 2019 - Mean (SE).

	Dependent variable:									
	Participation									
	2010	2011	2012	2013	2014	2015	2016	2017	2018	2019
IMD Score	−0.050∗**	−0.047∗**	−0.044∗**	−0.038∗**	−0.037∗**	−0.036∗**	−0.036∗**	−0.035∗**	−0.035∗**	−0.035∗**
	(0.0003)	(0.0002)	(0.0001)	(0.0001)	(0.0001)	(0.0001)	(0.0001)	(0.00005)	(0.00004)	(0.00004)
Ethnic-Density (%)	−0.014∗**	−0.013∗**	−0.012∗**	−0.013∗**	−0.013∗**	−0.012∗**	−0.012∗**	−0.011∗**	−0.010∗**	−0.009∗**
	(0.0002)	(0.0001)	(0.0001)	(0.0001)	(0.0001)	(0.00005)	(0.00004)	(0.00004)	(0.00003)	(0.00003)
Pop Density (sqkm)	−0.020∗**	−0.021∗**	−0.018∗**	−0.013∗**	−0.016∗**	−0.015∗**	−0.014∗**	−0.012∗**	−0.009∗**	−0.008∗**
	(0.001)	(0.001)	(0.0005)	(0.0003)	(0.0003)	(0.0002)	(0.0002)	(0.0002)	(0.0002)	(0.0001)
Distance (km)	−0.090∗**	−0.105∗**	−0.125∗**	−0.126∗**	−0.134∗**	−0.123∗**	−0.112∗**	−0.102∗**	−0.099∗**	−0.088∗**
	(0.0003)	(0.0003)	(0.0003)	(0.0002)	(0.0002)	(0.0002)	(0.0002)	(0.0002)	(0.0002)	(0.0001)
Constant	−3.430∗**	−2.939∗**	−2.497∗**	−1.996∗**	−1.642∗**	−1.482∗**	−1.317∗**	−1.204∗**	−1.158∗**	−1.039∗**
	(0.008)	(0.005)	(0.004)	(0.002)	(0.002)	(0.002)	(0.001)	(0.001)	(0.001)	(0.001)
Observations	32,844	32,844	32,844	32,844	32,844	32,844	32,844	32,844	32,844	32,844
Log Likelihood	−161,147.400	−283,456.700	−405,018.000	−613,713.800	−780,718.200	−889,065.000	−1,045,988.000	−1,161,922.000	−1,231,452.000	−1,393,382.000
Akaike Inf. Crit.	322,304.800	566,923.500	810,045.900	1,227,438.000	1,561,446.000	1,778,140.000	2,091,987.000	2,323,854.000	2,462,913.000	2,786,775.000

Note: Standard errors in parentheses *p < 0.1; ∗*p < 0.05; ∗**p < 0.01.

The Index of Multiple Deprivation regression coefficient is negative in every year over the ten-year period (i.e. more deprived areas have lower *parkrun* participation). However, the coefficient on IMD has increased throughout from −0.050 in 2010 to −0.035 in 2019, meaning the effect of a single unit increase in IMD score (controlling for covariates) changed from −5% in 2010 to −3.5% in 2019. Most of this change occurred between 2010 and 2013. It is also worth noting that the absolute value of the coefficient on the Ethnic Density variable has also increased over time. The effect of a 1% increase in ethnic density decreased from a 1.4% reduction in participation in 2010 to a 0.9% reduction in 2019. (i.e. the effect of ethnic density, the percentage of non-White-British persons in the community, on *parkrun* participation has fallen over time).

We also ran several additional analyses. Firstly we reproduce Table 3 with a quasi-poisson regression model, the results of which are shown in Table A3 in the appendix. Due to the high number of LSOAs which had no finishers in the earlier years (2010–2013), the standard errors are very large when using a quasi-poisson GLM model. We also ran the analyses separately for urban and rural areas. We found that the findings were consistent within urban areas (see Table A4 in the appendix). In rural areas, however, the relationship is less clear because there are few rural areas with high deprivation and high ethnic density. However, a similar trend can still be observed (see Table A5 in the appendix).

## 4 Discussion

This study investigates the trends in community level access to, and participation in *parkrun*, a community running and walking event in England between 2010 and 2019. Utilising the comprehensive datasets provided by *parkrun* and the ONS, we were able to show that distance to the nearest event decreased while participation increased over the ten year period. These can be considered as improvements given *parkrun*'s intention to improve geographical access and therefore participation. These improvements exhibited diminishing returns, however, with initial improvements being bigger than later improvements.

The distance to *parkrun* events was equal in 2010; *parkrun* events were situated at similar geodesic distances from more deprived areas and less deprived areas. However, as *parkrun* grew, more events were launched in areas with higher deprivation (mainly in cities) than in less deprived areas such as rural village locations. Perhaps because of these disproportionate reductions in distance for people from more deprived areas, participation among these communities also increased disproportionately until 2013 (Fig. 3, Fig. 4).

Despite *parkrun* events being situated closer to more deprived areas, we found a strong and persistent socioeconomic gradient in *parkrun* participation rates. This result was confirmed by the multivariable analysis of the determinants of *parkrun* participation, which showed only marginal changes in the relationship between IMD and *parkrun* participation after 2013 (from −0.039 to −0.036 in 2019; Table 3).

These findings are consistent with the results from a cross-section analysis from 2018, which showed that areas in England with higher deprivation and areas with higher ethnic density had lower *parkrun* participation rates (Schneider et al., 2020). In this paper we replicated this previous analysis for each year from 2010 to 2019. We found that, as with the descriptive statistics and univariable analysis (RII), the period can be split into two distinct phases: from 2010 to 2013 the effect of deprivation (IMD) on participation rates reduced, and from 2013 to 2019 the effect remained stable. However, the effect of ethnic density on participation appears to have declined over the entire period. *parkrun* is commonly held up as an example of a movement which is effective at increasing physical activity in the community (Reece et al., 2019; Stevinson et al., 2015). The events themselves have been perceived to be inclusive and sociable (Sharman et al., 2019; Hindley, 2020), and *parkrun* as an organisation has been particularly focused on making events accessible to everyone regardless of background and ability (Reece et al., 2019). One way in which *parkrun* has attempted to improve accessibility is through the creation of new events. This culminated in a partnership with Sport England in 2018 which aimed to create 200 new events targeted specifically towards socioeconomically deprived communities. Our previous work suggested that reduced distance to *parkrun* while likely to increase overall participation, may also widen pre-existing inequalities in participation (Smith et al., 2020; Schneider et al., 2020). This study validates these findings: mean distance to a *parkrun* event has consistently reduced and has reduced faster in more deprived areas, yet participation remains substantially lower compared to less deprived areas. It therefore seems unlikely that more events will substantially reduce inequalities in participation.

Further research is necessary to better understand why some communities are more engaged in *parkrun* than others. Understanding why engagement differs more or less at different times of the year may be a simple first step in this analysis, but a more robust mixed-methods approach to identifying modifiable factors which influence participation is more likely to generate feasible interventions. This could have a wider impact than just *parkrun*, since the mechanisms which affect participation in *parkrun* are also likely to influence physical activity participation and/or engagement in community events in general.

Our study fills an important gap in the literature as an exemplar of how community events which could increase physical activity can grow and adds to the understanding of how that growth occurs in different communities. Our findings have several implications for policy. Firstly, creating new events is likely to continue to increase overall participation in *parkrun*, but is unlikely to reduce the inequalities in participation that have been relatively constant for the past six years. Strategies to encourage engagement with socioeconomically deprived communities, such as considering transport methods for non-car users as suggested in Fullagar et al. (2019), could be incorporated into the creation of new events in order to maximize their impact, especially in socioeconomically deprived communities. Secondly, there does appear to be a trend of increasing engagement from areas with higher ethnic density. This is encouraging because it suggests that *parkrun* is becoming more successful at engaging with culturally diverse communities. *parkrun* could continue to promote participation in these communities, for example previous research has suggested engaging with community leaders or translating promotional materials into other languages (Fullagar et al., 2019).

Overall, our findings also suggest that the location of events may be less important than their social meaning, cultural relevance, and local perceptions of their accessibility– which has been discussed by other authors and supported in single UK localities (Jones et al., 2009; Macintyre, 2007). We speculate that the perceived accessibility of *parkrun* events contributes to the lower participation rates by people living in more deprived areas regardless of geographical distance. *parkrun* is just one form of outdoor, community-based physical activity that may have different appeal and accessibility to some groups more than others. Further research into *parkrun*, and with comparison to other outdoor community-based physical activity events is needed to understand the perceptions of accessibility and appeal held by local community members. This will help to understand whether accessibility issues relate to characteristics of the activity itself (e.g., type, time, location, duration) or wider social determinants of health and in turn, how these can be addressed to tackle inequalities and develop inclusive strategies to participation.

### 4.1 Limitations

The coefficients for 2018 do not perfectly match the coefficients of our previous paper (Schneider et al., 2020). There are several reasons for this: firstly, this analysis includes the full year, whereas the 2018 study included only the period to 10th December; secondly, *parkrun* updated their database, which led to some (seemingly) random variation between the two datasets, and finally we only include events held on a Saturday in this analysis, whereas in the previous analysis we included all *parkrun* events. This has no material impact on the findings or the implications for policy.

In this study, we used distance to the nearest *parkrun* event as a proxy for access. However, geographic distance may be a sub-optimal measure of the ability of different groups to attend events. A 5 km distance may be more difficult to transverse in a city than for those with a car in rural areas. A model which uses estimates of travel time using travel distance and predicted transport mode may yield a better proxy for travel access, and adding a consideration of other forms of perceived access (e.g. travel expense, safety) may improve our understanding of the determinants of participation.

The use of ethnic density in this analysis does not allow us to determine variation in participation by areas with higher percentages of specific ethnic groups. Future analysis could use more detailed ONS data on the LSOAs to better understand whether the effect is similar for all minority ethnic groups. All data and code is available open source to enable others to build upon this work.

There are also several limitations that are similar from our previous analysis for 2018 (Schneider et al., 2020). Firstly, the data provided by *parkrun* gives the number of finishes by LSOA. The number of finishes tells us little about the number of unique runners, and one runner undertaking 5 runs is counted the same as five runners undertaking 1 run each. We feel confident this is a satisfactory simplification. We also include only those who finish the *parkrun* and scan their barcode as participants. Feedback from *parkrunUK* suggests that the vast majority (>90%) of participants do finish the 5 km walk/run and scan their barcode. However small biases may exist if those from areas with higher levels of socioeconomic deprivation are less likely to finish or scan their code.

It is also important to note that this study is an ecological study at the level of the LSOA, and all findings have been discussed at the level of the community rather than the individual, so as to avoid an ecological inference fallacy. Future research, with access to more detailed data on the characteristics of individual *parkrun* participants, may be able to better understand individual-level drivers on participation.

Finally, as a walking and running event, *parkrun* is not representative of all types of physical activity. It may be the case that different communities, or even sub-groups within communities, would engage differently to other types of events. Since we do not have data on *parkrun* participation by gender it is not possible to know if this is an important factor. Future studies should attempt to obtain data on participation split by gender to determine whether the socioeconomic factors influence male and female participation differently.

## 5 Conclusion

Over the past 10 years, geodesic distances to the nearest *parkrun* decreased from a mean of 34 km to5 km. In 2010, there was equality between the least and most deprived areas but by 2017 the distance of the most deprived areas was 29% that of the least deprived. Participation was shown to have increased over the past 10 years which can be split into two distinct phases: from 2010 to 2013 participation increased super-linearly and inequality in participation fell dramatically; from 2013 to 2019 participation increased linearly, and inequality in participation remained stable.

The findings of this study suggest that, by 2019, *parkrun* had reached a steady (linear) rate of growth in participation and the share of participation by different socioeconomic groups (e.g. quintiles of IMD). While participation is likely to continue to increase for all socioeconomic groups, closing the gap in participation between the most and least deprived communities is likely to require changes to the organisation and delivery of events rather than just further increases in the number of events in more deprived areas.

Mixed methods research combining the power of the rich participation dataset provided by *parkrun* with a deeper understanding of the issues on the ground is essential for shaping effective interventions to boost participation overall, but particularly in socio-economically deprived communities.

## Declaration of competing interest

R.S. and P.S. have no competing interests. S.H. is chair, A.B. and H.Q are deputy chairs, and L.G. is a member of the parkrun research board.

## CRediT authorship contribution statement

**Robert A. Smith:** Conceptualization, Data curation, Formal analysis, Investigation, Methodology, Project administration, Software, Visualization, Writing – original draft, Preparation, Writing – review & editing. **Paul P. Schneider:** Conceptualization, Data curation, Formal analysis, Investigation, Methodology, Project administration, Software, Visualization, Writing – original draft, Preparation, Writing – review & editing. **Rami Cosulich:** Validation, Writing – review & editing, Writing – original draft, Preparation. **Helen Quirk:** Writing – review & editing, Writing – original draft, Preparation. **Alice M. Bullas:** Project administration, Resources. **Steve J. Haake:** Project administration, Resources, Writing – review & editing. **Elizabeth Goyder:** Supervision, Writing – review & editing.

## Figures and Tables

**Table A1:** Mean geodesic distance in km to the nearest *parkrun* event by IMD quintile for each year from 2010 to 2019.

Year	2010	2011	2012	2013	2014	2015	2016	2017	2018	2019
Overall	34.09 (0.06)	20.79 (0.04)	14.53 (0.03)	10.21 (0.02)	7.93 (0.01)	6.66 (0.01)	5.79 (0.01)	5.27 (0.01)	4.85 (0.01)	4.58 (0.01)
Most deprived 20%	30.57 (0.13)	16.84 (0.08)	11.21 (0.05)	7.72 (0.03)	6.02 (0.02)	5.03 (0.02)	4.29 (0.01)	3.86 (0.01)	3.6 (0.01)	3.46 (0.01)
2	35.64 (0.17)	21.52 (0.11)	14.86 (0.07)	10.19 (0.05)	7.72 (0.03)	6.36 (0.02)	5.35 (0.02)	4.82 (0.02)	4.43 (0.02)	4.2 (0.02)
3	38.82 (0.15)	23.94 (0.10)	16.9 (0.07)	11.95 (0.04)	9.18 (0.03)	7.77 (0.03)	6.73 (0.02)	6.05 (0.02)	5.61 (0.02)	5.25 (0.02)
4	35.52 (0.13)	22.2 (0.08)	15.7 (0.06)	11.12 (0.04)	8.61 (0.03)	7.29 (0.02)	6.39 (0.02)	5.88 (0.02)	5.44 (0.02)	5.13 (0.01)
Least deprived 20%	29.92 (0.10)	19.46 (0.06)	13.98 (0.04)	10.09 (0.03)	8.11 (0.02)	6.85 (0.02)	6.18 (0.02)	5.73 (0.02)	5.16 (0.01)	4.84 (0.01)

1 = most socioeconomically deprived quintile, 5 = least socioeconomically deprived quintile, Standard errors in parentheses.

**Table 2:** Mean monthly *parkrun* finishers per 1000 persons by IMD quintile for each year from 2010 to 2019.

Year	2010	2011	2012	2013	2014	2015	2016	2017	2018	2019
Overall	0.26 (0.00)	0.6 (0.00)	1.23 (0.00)	2.71 (0.01)	4.38 (0.01)	6.13 (0.01)	8.26 (0.02)	10.43 (0.02)	11.8 (0.02)	14.43 (0.02)
Most deprived 20%	0.07 (0.00)	0.18 (0.00)	0.42 (0.00)	1 (0.01)	1.61 (0.01)	2.21 (0.01)	2.97 (0.01)	3.82 (0.02)	4.3 (0.02)	5.22 (0.02)
2	0.15 (0.00)	0.37 (0.00)	0.81 (0.01)	1.93 (0.01)	3.14 (0.02)	4.32 (0.02)	5.85 (0.03)	7.53 (0.03)	8.66 (0.03)	10.52 (0.04)
3	0.21 (0.00)	0.52 (0.01)	1.1 (0.01)	2.54 (0.02)	4.24 (0.02)	5.89 (0.03)	8.12 (0.03)	10.37 (0.04)	11.83 (0.04)	14.59 (0.05)
4	0.34 (0.01)	0.77 (0.01)	1.55 (0.01)	3.37 (0.02)	5.58 (0.03)	7.87 (0.03)	10.65 (0.04)	13.35 (0.04)	15.08 (0.05)	18.51 (0.06)
Least deprived 20%	0.54 (0.01)	1.17 (0.01)	2.28 (0.02)	4.69 (0.03)	7.35 (0.03)	10.36 (0.04)	13.72 (0.05)	17.07 (0.05)	19.14 (0.05)	23.32 (0.06)

1 = most socioeconomically deprived quintile, 5 = least socioeconomically deprived quintile, Standard errors in parentheses.

**Table A3:** Results of the quasipoisson log-link generalised linear model for each year from 2010 to 2019.

	*Dependent variable:*									
	Participation									
	2010	2011	2012	2013	2014	2015	2016	2017	2018	2019
IMD Score	−0.050	−0.047	−0.044	−0.038***	−0.037***	−0.036***	−0.036***	−0.035***	−0.035***	−0.035***
	(6.619)	(0.356)	(0.166)	(0.002)	(0.001)	(0.0005)	(0.0004)	(0.0004)	(0.0004)	(0.0004)
Ethnic-Density (%)	−0.014	−0.013	−0.012	−0.013v**	−0.013***	−0.012***	−0.012***	−0.011***	−0.010***	−0.009***
	(4.328)	(0.245)	(0.117)	(0.002)	(0.001)	(0.0004)	(0.0003)	(0.0003)	(0.0003)	(0.0003)
Pop Density (sqkm)	−0.020	−0.021	−0.018	−0.013*	−0.016***	−0.015***	−0.014***	−0.012***	−0.009***	−0.008***
	(19.129)	(1.114)	(0.538)	(0.008)	(0.003)	(0.002)	(0.002)	(0.001)	(0.001)	(0.001)
Distance(km)	−0.090	−0.105	−0.125	−0.126***	−0.134***	−0.123***	−0.112***	−0.102***	−0.099***	−0.088***
	(6.908)	(0.528)	(0.342)	(0.005)	(0.002)	(0.001)	(0.001)	(0.001)	(0.001)	(0.001)
Constant	−3.430	−2.939	−2.497	−1.996***	−1.642***	−1.482***	−1.317***	−1.204***	−1.158***	−1.039***
	(149.760)	(8.706)	(4.214)	(0.059)	(0.019)	(0.012)	(0.011)	(0.010)	(0.010)	(0.009)
Observations	32,844	32,844	32,844	32,844	32,844	32,844	32,844	32,844	32,844	32,844

Note: Standard errors in parentheses *p < 0.1; ∗*p < 0.05; ∗**p < 0.01.

Note the high standard errors for the quasi-poisson regression model due to the relatively low number of LSOAs with non-zero finishes.

**Table A4:** Results of the Poisson log-link generalised linear model for each year from 2010 to 2019 - URBAN LOCATIONS ONLY

	*Dependent variable:*									
	Participation									
	2010	2011	2012	2013	2014	2015	2016	2017	2018	2019
IMD Score	−0.051***	−0.049***	−0.044***	−0.039***	−0.038***	−0.037***	−0.037***	−0.037***	−0.036***	−0.036***
	(0.0003)	(0.0002)	(0.0001)	(0.0001)	(0.0001)	(0.0001)	(0.0001)	(0.00005)	(0.00005)	(0.00004)
Ethnic-Density (%)	−0.016***	−0.015***	−0.012***	−0.013***	−0.013***	−0.013***	−0.012***	−0.011***	−0.010***	−0.009***
	(0.0002)	(0.0001)	(0.0001)	(0.0001)	(0.0001)	(0.00005)	(0.00004)	(0.00004)	(0.00003)	(0.00003)
Pop Density (sqkm)	−0.022***	−0.020***	−0.017***	−0.010***	−0.011***	−0.009***	−0.008***	−0.006***	−0.004***	−0.002***
	(0.001)	(0.001)	(0.0005)	(0.0003)	(0.0003)	(0.0002)	(0.0002)	(0.0002)	(0.0002)	(0.0001)
Distance (km)	−0.104***	−0.124***	−0.148***	−0.148***	−0.164***	−0.154***	−0.147***	−0.139***	−0.141***	−0.131***
	(0.0004)	(0.0004)	(0.0004)	(0.0003)	(0.0003)	(0.0003)	(0.0002)	(0.0002)	(0.0002)	(0.0002)
Constant	−3.238***	−2.767***	−2.357***	−1.895***	−1.546***	−1.394***	−1.227***	−1.110***	−1.047***	−0.934***
	(0.008)	(0.005)	(0.004)	(0.003)	(0.002)	(0.002)	(0.002)	(0.001)	(0.001)	(0.001)
Observations	27,246	27,246	27,246	27,246	27,246	27,246	27,246	27,246	27,246	27,246
Log Likelihood	−136,070.000	−237,138.600	−338,896.800	−510,965.600	−636,803.900	−724,510.800	−845,411.200	−940,332.100	−998,117.900	−1,129,960.000
Akaike Inf. Crit.	272,150.100	474,287.300	677,803.600	1,021,941.000	1,273,618.000	1,449,032.000	1,690,832.000	1,880,674.000	1,996,246.000	2,259,929.000

Note: Standard errors in parentheses *p < 0.1; ∗*p < 0.05; ***p < 0.01.

**Table A5:** Results of the Poisson log-link generalised linear model for each year from 2010 to 2019 - RURAL LOCATIONS ONLY

	*Dependent variable:*									
	Participation									
	2010	2011	2012	2013	2014	2015	2016	2017	2018	2019
IMD Score	−0.027***	−0.024***	−0.030***	−0.023***	−0.023***	−0.024***	−0.025***	−0.025***	−0.025***	−0.025***
	(0.001)	(0.001)	(0.001)	(0.0004)	(0.0003)	(0.0002)	(0.0002)	(0.0002)	(0.0002)	(0.0001)
Ethnic-Density (%)	0.028***	0.007***	−0.005***	−0.016***	−0.028***	−0.022***	−0.021***	−0.019***	−0.019***	−0.017***
	(0.002)	(0.001)	(0.001)	(0.001)	(0.001)	(0.0004)	(0.0004)	(0.0003)	(0.0003)	(0.0003)
Pop Density (sqkm)	−0.076***	−0.054***	−0.028***	−0.053***	−0.061***	−0.061***	−0.065***	−0.065***	−0.064***	−0.076***
	(0.010)	(0.006)	(0.004)	(0.002)	(0.002)	(0.002)	(0.001)	(0.001)	(0.001)	(0.001)
Distance (km)	−0.038***	−0.056***	−0.077***	−0.089***	−0.100***	−0.095***	−0.088***	−0.080***	−0.076***	−0.066***
	(0.001)	(0.001)	(0.0005)	(0.0004)	(0.0004)	(0.0003)	(0.0003)	(0.0003)	(0.0003)	(0.0002)
Constant	−5.171***	−4.064***	−3.163***	−2.386***	−1.833***	−1.622***	−1.407***	−1.277***	−1.238***	−1.104***
	(0.030)	(0.018)	(0.013)	(0.008)	(0.006)	(0.005)	(0.004)	(0.004)	(0.004)	(0.003)
Observations	5598	5598	5598	5598	5598	5598	5598	5598	5598	5598
Log Likelihood	−21,664.750	−40,958.460	−59,150.570	−92,296.000	−124,148.400	−141,736.200	−169,912.100	−186,956.600	−193,729.400	−217,554.900
Akaike Inf. Crit.	43,339.490	81,926.930	118,311.100	184,602.000	248,306.700	283,482.400	339,834.100	373,923.300	387,468.800	435,119.800

Note: Standard errors in parentheses *p < 0.1; **p < 0.05; ***p < 0.01.

^1^Note that we use thousand persons per km^2^ here to enable easier interpretation of the regression coefficients.
